# Medium Effects on Minimum Inhibitory Concentrations of Nylon-3 Polymers against *E. coli*

**DOI:** 10.1371/journal.pone.0104500

**Published:** 2014-08-25

**Authors:** Heejun Choi, Saswata Chakraborty, Runhui Liu, Samuel H. Gellman, James C. Weisshaar

**Affiliations:** 1 Department of Chemistry, University of Wisconsin-Madison, Madison, Wisconsin, United States of America; 2 Molecular Biophysics Program, University of Wisconsin-Madison, Madison, Wisconsin, United States of America; University Hospital Schleswig-Holstein, Campus Kiel, Germany

## Abstract

Minimum inhibitory concentrations (MICs) against *E. coli* were measured for three nylon-3 polymers using Luria-Bertani broth (LB), brain-heart infusion broth (BHI), and a chemically defined complete medium (EZRDM). The polymers differ in the ratio of hydrophobic to cationic subunits. The cationic homopolymer is inert against *E. coli* in BHI and LB, but becomes highly potent in EZRDM. A mixed hydrophobic/cationic polymer with a hydrophobic *t*-butylbenzoyl group at its N-terminus is effective in BHI, but becomes more effective in EZRDM. Supplementation of EZRDM with the tryptic digest of casein (often found in LB) recapitulates the LB and BHI behavior. Additional evidence suggests that polyanionic peptides present in LB and BHI may form electrostatic complexes with cationic polymers, decreasing activity by diminishing binding to the anionic lipopolysaccharide layer of *E. coli*. In contrast, two natural antimicrobial peptides show no medium effects. Thus, the use of a chemically defined medium helps to reveal factors that influence antimicrobial potency of cationic polymers and functional differences between these polymers and evolved antimicrobial peptides.

## Introduction

There is a pressing need to develop new ways to kill harmful bacteria while causing minimal damage to eukaryotic cells [1], [2]. An important component of the innate response to invasive bacteria is the release of antimicrobial peptides (AMPs) that permeabilize bacterial membranes and ultimately kill the invaders [3]–[5]. These peptides target bacterial membranes selectively relative to eukaryotic membranes. Thousands of natural AMPs are now known [6]. Substantial effort has been devoted to development of synthetic analogues containing α-amino acid residues and/or unnatural subunits that mimic the selective antibacterial action of AMPs. Examples include discrete oligomers generated from L-α-amino acids [7], [8], N-alkyl glycines (“peptoids”) [9]–[11], β-amino acids (“β-peptides”) [12], [13], or combinations of these building blocks. Many of these oligomers have been designed to adopt an amphipathic helical conformation, because this structural motif is common among natural AMPs.

The synthesis of sequence-specific oligomers requires a step-by-step approach, typically involving solid-phase methods, which is time-consuming and expensive. This synthetic problem has encouraged several research groups to explore polymerization-based methods to generate antimicrobial materials [14]–[22]. In many cases, a pair of precursors is copolymerized, with one precursor giving rise to a hydrophobic subunit and the other giving rise to a cationic subunit in the polymer chains. The resulting materials are heterogeneous, containing chains that vary in length, composition, subunit sequence and, frequently, subunit stereochemistry. Antibacterial activity in such cases cannot depend on adoption of a single amphipathic conformation. Nevertheless, careful tuning of the proportion and identities of the cationic and hydrophobic subunits can provide polymeric materials that exhibit strong bacteriostatic action against both Gram negative and Gram positive bacteria at concentrations that do not cause destruction of red blood cells (“hemolysis”).

Our recent structure-function study of binary nylon-3 copolymers (β-peptide backbone) showed that a specific proportion of hydrophobic and cationic subunits plus inclusion of a hydrophobic group such as p-*t*-butylbenzoyl at the N-terminus provided a favorable balance of bacteriostatic and hemolytic properties [19]. Bacteriostatic potency was evaluated in terms of the minimum inhibitory concentration (MIC), the lowest polymer concentration that halted bacterial growth. Four bacterial species were evaluated, among which *Escherichia coli* was the only Gram negative organism. *E. coli* MIC measurements were carried out using brain-heart infusion (BHI) growth medium. Hemolytic activity was measured as the minimum hemolytic concentration (MHC), the smallest polymer concentration that caused detectable release of hemoglobin from human red blood cells.

We have been developing fluorescence microscopy methods that monitor membrane disruption induced by antimicrobial peptides acting on single bacterial cells in real time [23]–[25]. The broths typically used for rapid bacterial growth, including brain-heart infusion (BHI) and Luria-Bertani (LB), are unsuitable for sensitive fluorescence work due to their strong background fluorescence on excitation with visible light. Instead, we use a low-fluorescence, chemically defined medium called “EZ rich, defined medium” (EZRDM) [26]. As a prelude to studies of the mechanism of nylon-3 action against *E. coli*, we measured MIC values for a panel of nylon-3 polymers in the EZRDM medium. Surprisingly, we observed a dramatic reduction of MIC values (greater polymer efficacy) in EZRDM as compared with either BHI or LB.

In particular, cationic homopolymers (lacking hydrophobic subunits) were much more effective against *E. coli* in EZRDM than in BHI or LB media.

We report studies intended to elucidate the effect of bacterial growth medium on MIC values measured for *E. coli*. By adding tryptone, the tryptic digest of bovine casein, to EZRDM, we recover the pattern of activity observed in BHI. We also present evidence indicating that anionic peptide components of tryptone (and, by extension, those in BHI and LB) diminish the ability of the highly cationic polymers to attack *E. coli* relative to the effects observed in EZRDM. This functional attenuation presumably results from formation of relatively inert electrostatically bound complexes between the cationic polymers and the anionic peptide components. In sharp contrast to the behavior of the cationic polymers, two natural AMPs (LL-37 and Cecropin A) showed consistent MIC values in all media. Another natural AMP, Magainin 2, was inactive against *E. coli* in EZRDM and had low activity in BHI as well. We suggest that the MIC measurements in EZRDM reveal the “intrinsic activity” of a polymer or AMP.

These results expand our understanding of structure-activity relationships among the nylon-3 polymers and suggest new design strategies for the future. Moreover, these observations highlight a previously undocumented feature of natural AMPs, which can apparently be fine-tuned by evolutionary selection to avoid the polyanion-based neutralization mechanism suggested by our polymer findings.

## Materials and Methods

### Materials

**Figure 1:**
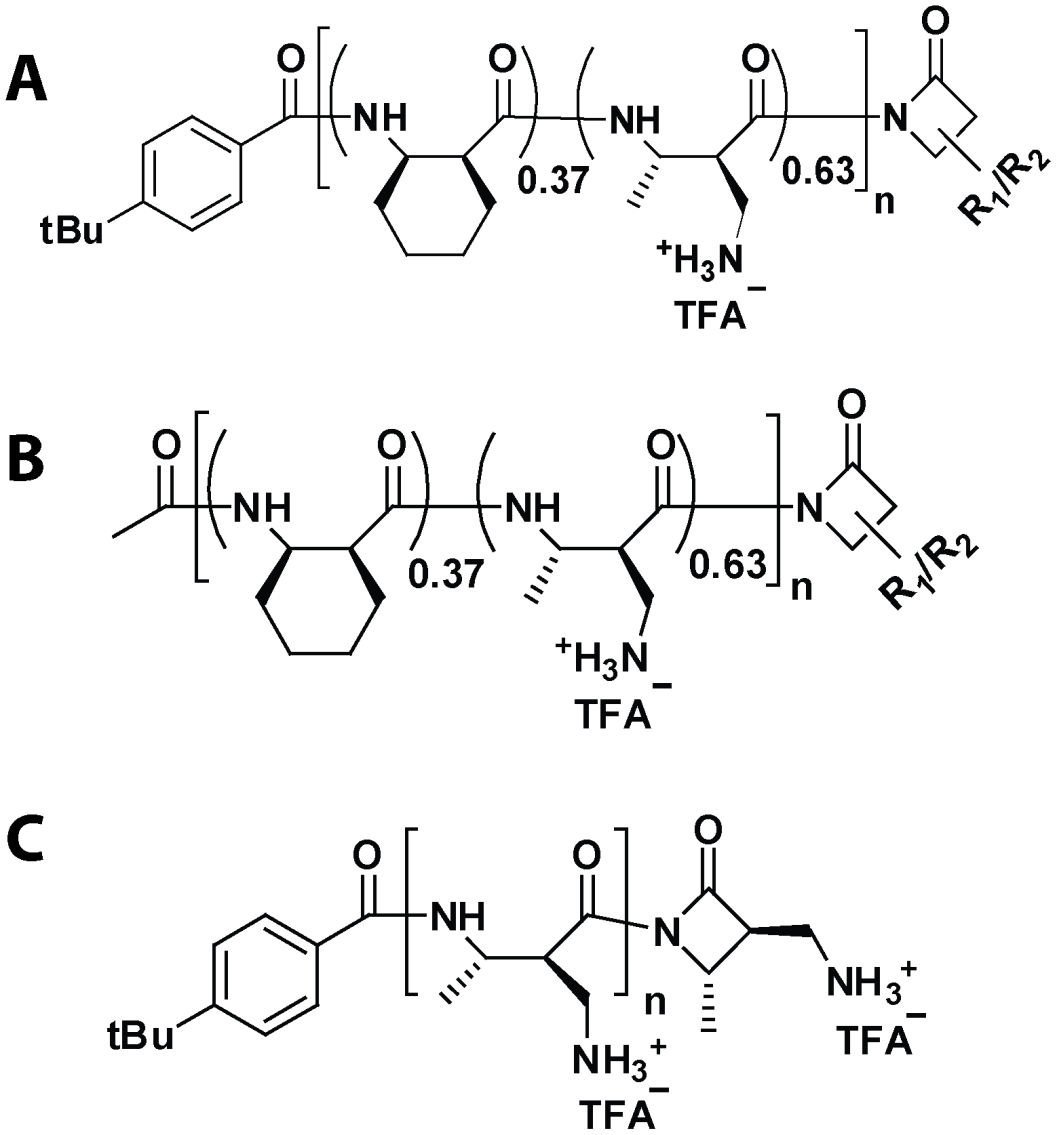
Structures of the random, heterochiral nylon-3 polymers used in this study. **A** bears a hydrophobic p-*t*-butylbenzoyl group at the N-terminus. **B** shares the 37∶63 CH:MM ratio of **A**, but lacks a hydrophobic group at the N-terminus. **C** is a homopolymer of the cationic MM subunit.

The nylon-3 polymers used in this study (Fig. 1) were synthesized as previously described [19]. Polymers were prepared from racemic β-lactams, and each polymer was therefore heterochiral. Polymer samples had mean chain lengths of 27 (polymer **A**), 27 (**B**), and 24 (**C**). Polydispersity index (PDI) values ranged from 1.02–1.15. To check for possible batch-to-batch variation in polymer properties, each polymer was synthesized twice. The MIC values measured for different batches of the same polymer were indistinguishable in each case. Human cathelicidin LL-37 was purchased from Bachem, and moth Cecropin A and Magainin 2 were purchased from Anaspec. All three peptides were used without purification. EZ rich defined medium (EZRDM, Teknova), brain heart infusion broth (BHI, Difco), and Luria-Bertani broth (LB, Sigma Aldrich) were purchased as powders and dissolved in water. Tryptone powder was purchased from BD Sciences. We compared the effects of supplements made from tryptone powder as received vs tryptone powder that was dissolved, dialyzed to remove small ions and solutes (1 kDa cutoff), and then lyophilized. No differences were observed. We use “tryptone” or “dialyzed tryptone” to mean a solution of the dialyzed tryptone powder dissolved in EZRDM. We use “raw tryptone” to mean a solution of the tryptone powder as received. The free base ion-exchange resin Amberlite IRA67 was purchased from Sigma Aldrich.

We devised two tests of the effects of anionic species within tryptone on MIC values. First, the large polyanions in raw tryptone were removed from dialyzed tryptone solution using a washed, free-base anion-exchange resin (Amberlite IRA67, Sigma Aldrich). At pH = 7, the resin removes H^+^ and polyanions from solution. A solution of dialyzed tryptone was incubated with 1.2X (wt/wt) resin for 2 hr and then filtered to remove the beads. We designate the product of this operation “anion-exchanged tryptone”. The filtered product was lyophilized and added to EZRDM for MIC assays. Second, we used solid-phase methods to synthesize a single, specific anionic peptide, FQSEEQQTEDELQDK, and tested its effects at 400 µM in EZRDM.

This peptide is a putative component of raw tryptone based on the predicted products of digestion of bovine beta-casein by trypsin. The peptide was used without purification (estimated purity ∼85%).

### Minimum Inhibitory Concentration (MIC) Assay

*E. coli* strains JM109 and MG1655 were studied. MIC values were determined using a standard serial microdilution method. Serial two-fold dilutions of each nylon-3 polymer, and of LL-37, Cecropin A, and Magainin 2, were performed in separate rows of a polystyrene 96-well plate in the chosen medium containing an inoculum of either JM109 or MG1655. Polymer concentrations between 6.3 µg/mL and 200 µg/mL were evaluated. Each assay plate contained a dilution series with ampicillin as a positive control. To test for possible effects of the MIC procedural details, the measurements in BHI medium were carried out with cells initially sampled either from stationary phase (as in the earlier study) [19] or from mid-log phase. In the stationary phase procedure, a culture grown overnight at 37°C to stationary phase was sampled and diluted to OD_600_ = 0.05 with medium at the same temperature. In the mid-log phase procedure, the stationary culture was diluted in fresh medium (1∶100) and grown until it reached OD_600_ = 0.5. For the MIC measurements, the plate was incubated at 37°C for 6 hr. For both the stationary phase procedure and the mid-log phase procedure, we tested for effects of stationary incubation in a VWR 1525 digital incubator vs increased aeration due to shaking at 200 rpm in a Lab-Line Orbital Environ Shaker (Model 3527). For experiments augmenting EZRDM medium with tryptone solution, the appropriate amount of raw or dialyzed tryptone powder was dissolved in EZRDM and used in the dilution steps.

The MIC results were not affected by the choice of *E. coli* strain or any of the other variations in experimental procedure. Following these initial tests, our standard procedure used stationary phase cultures with no shaking during outgrowth and tryptone solution made from dialyzed tryptone powder. The overnight culture of *E. coli* was grown in EZRDM prior to the addition of tryptone supplemented EZRDM.

**Figure 2:**
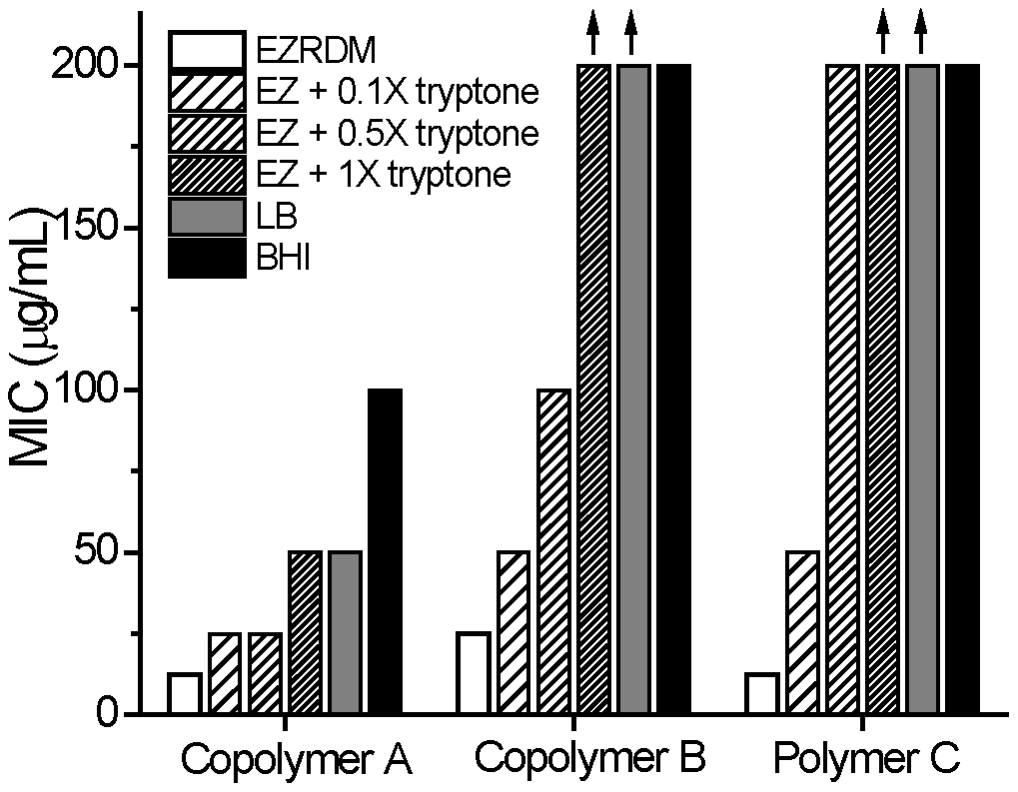
Minimum inhibitory concentrations (MICs) of three nylon-3 polymers against *E. coli* for different media. See Fig. 1 for structures of **A**, **B**, and **C**. Vertical arrows mark bars that are lower limits only. EZRDM, LB, and BHI as described in main text. The designation “EZ + trypt” refers to EZRDM supplemented with 10 g/L of dialyzed tryptone powder (1X tryptone). “EZ + trypt – PA” refers to EZRDM supplemented with anion-exchanged tryptone at the equivalent of 10 g/L. “EZ + FQS…” refers to EZRDM supplemented with 400 µM of the single anionic peptide FQSEEQQTEDELQDK (net –5 charge).

The MIC is reported as the lowest concentration for which no cell growth could be detected after 6 hr (OD = 0.00±0.05), as determined by measurements at 595 nm using an EnVision 2100 Multilabel Reader (Perkin-Elmer). Examples of OD vs time curves are provided in Figs. S1, S2, and S3. With care, the resulting MIC measurements are more accurate than a factor of two, as evidenced by exact reproduction over repeated trials of the particular concentration step that halted growth over 6 hr. When growth inhibition did not occur at the highest concentration of polymer studied (200 µg/mL), we report 200 µg/mL as a lower bound on the MIC, denoted by an upward arrow in the MIC bar graphs (Fig. 2).

## Results

### Experimental Design

This study focuses on three nylon-3 polymers that have been previously described [19] (Fig. 1; **A–C**). **A** is a copolymer generated from a ring-opening polymerization reaction mixture containing two β-lactams, CHβ and MMβ, in 37∶63 molar ratio. “CH” denotes a hydrophobic cyclohexyl side chain within the monomer and “MM” (monomethyl) denotes a single methyl group at the C_α_ site of the cationic monomer. Since the β-lactams are racemic, the resulting polymer sample contains chains with many different stereochemistries, as well as many different sequences. After polymerization, deprotection provides a side chain amino group in the MM subunit. These amino groups are protonated when the polymer is dissolved in aqueous solution near or below neutral pH, which confers a positive charge on the polymer.

The 37∶63 ratio of CH and MM subunits was previously shown [19] to provide a favorable compromise between antibacterial activity in BHI medium (lower MIC preferred) and hemolytic activity (higher minimum hemolytic concentration, MHC, preferred). Antibacterial activity in BHI medium was enhanced by the presence of a hydrophobic group at the N-terminus of the polymer chains; **A** bears a p-*t*-butylbenzoyl group at this position. In BHI medium, nylon-3 polymers **B** and **C** showed diminished antibacterial activity relative to **A**. Copolymer **B** shares the 37∶63 CH:MM ratio of **A**, but **B** lacks a hydrophobic group at the N-terminus. **C** is a homopolymer of the cationic MM subunit.

We were originally motivated to explore cationic-hydrophobic copolymers such as **A** because AMPs are commonly rich in both cationic and hydrophobic amino acid residues. A net positive charge is believed to be necessary to attract AMPs to the lipopolysaccharide (LPS) surface of a bacterial cell, which bears a net negative charge. Hydrophobic side chains are thought to be essential for interaction with the nonpolar interior of a lipid bilayer and the resulting membrane barrier disruption. From this perspective, it is sensible that a cationic homopolymer such as **C** would not display strong antibacterial effects, as observed in previous studies of *E. coli* MICs conducted in BHI medium [19]. We were therefore surprised to discover that cationic homopolymer **C** is highly active against *E. coli* in the chemically defined EZRDM. The experiments described here were undertaken to try to determine why MIC results differ between a traditional but chemically undefined medium such as BHI and chemically defined EZRDM.

EZRDM is a MOPS-buffered solution that contains glucose (0.2%), supplemental amino acids and vitamins, nucleotides, 1.32 mM K_2_HPO_4_, and 76 mM NaCl. This is our preferred medium for optical imaging experiments on bacteria because of its low autofluorescence [26].

In contrast, BHI medium is not chemically defined, since it is generated from calf brain and heart. BHI medium is supplemented with peptone, a digest of an undefined mixture of proteins from cow and pig. The enzyme used in the digest is proprietary, and the peptide mixture in peptone is therefore uncharacterized. As shown below, we found that *E. coli* MIC studies conducted in LB provide results similar to those conducted in BHI. LB is a common bacterial growth medium that, like BHI medium, is chemically undefined. LB medium contains tryptone along with yeast extract and sodium chloride. Tryptone is generated via tryptic digestion of casein.

We considered the hypothesis that peptides from a supplement such as tryptone (an additive in LB) or peptone (an additive in BHI medium) might interact with cationic nylon-3 polymers in a way that affects antibacterial potency. Since EZRDM contains amino acids rather than enzymatically generated peptides, the peptide-polymer interactions that we proposed to inhibit antibacterial activity of nylon-3 polymers would not be possible in EZRDM. Thus, the absence of enzymatically generated peptides in EZRDM might explain why polymer MIC values were lower in this medium than in LB or BHI medium.

This hypothesis was tested by supplementing EZRDM with dialyzed tryptone solution.

The supplement was generated by dialyzing raw tryptone solution against pure water for 48 hr to remove small ions and molecules below 1000 Da. The retained material was assumed to be composed largely of peptides generated via the cleavage of casein by trypsin. The retained solution was lyophilized, and the resulting dialyzed tryptone powder was added in varying proportions to EZRDM. As shown by the data presented below, this simple supplementation strategy caused a shift in nylon-3 MIC values for *E. coli* from the EZRDM profile to the profile observed in complex media such as BHI or LB.

Two additional sets of measurements tested whether or not anionic peptides were responsible for the larger MIC values observed on addition of dialyzed tryptone to EZRDM.

We measured MICs against *E. coli* for copolymers **A**, **B**, and **C** in EZRDM supplemented by the single anionic peptide FQSEEQQTEDELQDK. We also measured MICs for **A**, **B**, and **C** in EZRDM supplemented with anion-exchanged tryptone, which should lack anionic polypeptides. Both tests support the hypothesis that complexation of the highly cationic copolymer with polyanionic peptides diminishes antimicrobial activity.

### MIC measurements for polymers

Results obtained for the nylon-3 polymers in BHI medium (Fig. 2) agree with previous reports: cationic-hydrophobic copolymer **A** displays moderate activity against *E. coli* in this medium, but absence of the hydrophobic N-terminal group (copolymer **B**) or absence of the hydrophobic CH subunits (homopolymer **C**) leads to a profound loss of activity. MIC values measured in LB medium show the same pattern as those measured in BHI medium (Fig. 2).

In contrast, the MIC pattern in EZRDM is quite different: all three polymers are more active in EZRDM than in BHI or LB, and there is little distinction among the three polymers in EZRDM.

We supplemented EZRDM with dialyzed tryptone solution at three concentrations, 1 g/L, 5 g/L or 10 g/L, to generate media designated “EZ + 0.1X tryptone”, “EZ + 0.5X tryptone” and “EZ + 1X tryptone”. These designations are based on the fact that LB medium typically contains 10 g of tryptone powder per liter. Detailed MIC curves and MIC values vs tryptone concentrations are included in Figs. S3A and B. The antimicrobial activity of each polymer decreases (i.e., MIC value increases) as the concentration of dialyzed tryptone in EZRDM increases. The effects of 1X tryptone supplement on the MIC values for the three polymers are included in Fig. 2. The factor by which the MIC increases depends on polymer composition: polymer **A**, which is the most active of the three in complex media, is less strongly affected than polymers **B** and **C**, both of which are inactive in complex media but highly active (low MIC values) in EZRDM. Supplementation of EZRDM with 1X dialyzed tryptone recapitulates the effects of BHI and LB on MIC values. Polymer **A**, the most active polymer in BHI and LB, is the only polymer that retains reasonably good activity in EZRDM supplemented with 1X tryptone, in BHI, and in LB.

To test the ability of polyanions within dialyzed tryptone to alter MICs, we supplemented EZRDM with anion-exchanged tryptone, from which polyanionic components have been removed. The concentration of anion-exchanged tryptone was 10 g/L (as in 1X tryptone) *minus* the mass of the polyanions removed by the resin. The effects on MIC values for the three polymers are summarized in Fig. 2. Removal of polyanions from tryptone drastically reduces the MICs of the three polymers to the point where they are comparable to the MICs in EZRDM.

In addition, the pattern of relative MICs in EZRDM is recovered by removal of anionic molecules from the tryptone additive.

To investigate further the effect of polyanionic peptides in tryptone, the anionic peptide FQSEEQQTEDELQDK was added to EZRDM without tryptone supplementation. The anionic peptide concentration was chosen as 200 µM or 400 µM. The value 400 µM matches the estimated concentration of this particular peptide in 1X tryptone (based on 10 g/L of the digest products of the 23 kDa protein bovine beta-casein). The MICs of all three polymers increased with added concentration of the anionic peptide, as shown in Fig. S5. Addition of 400 µM of added anionic peptide increases the MICs of all three polymers by a factor of four to eight compared with EZRDM (Fig. 2).

### MIC measurements for antimicrobial peptides

**Figure 3:**
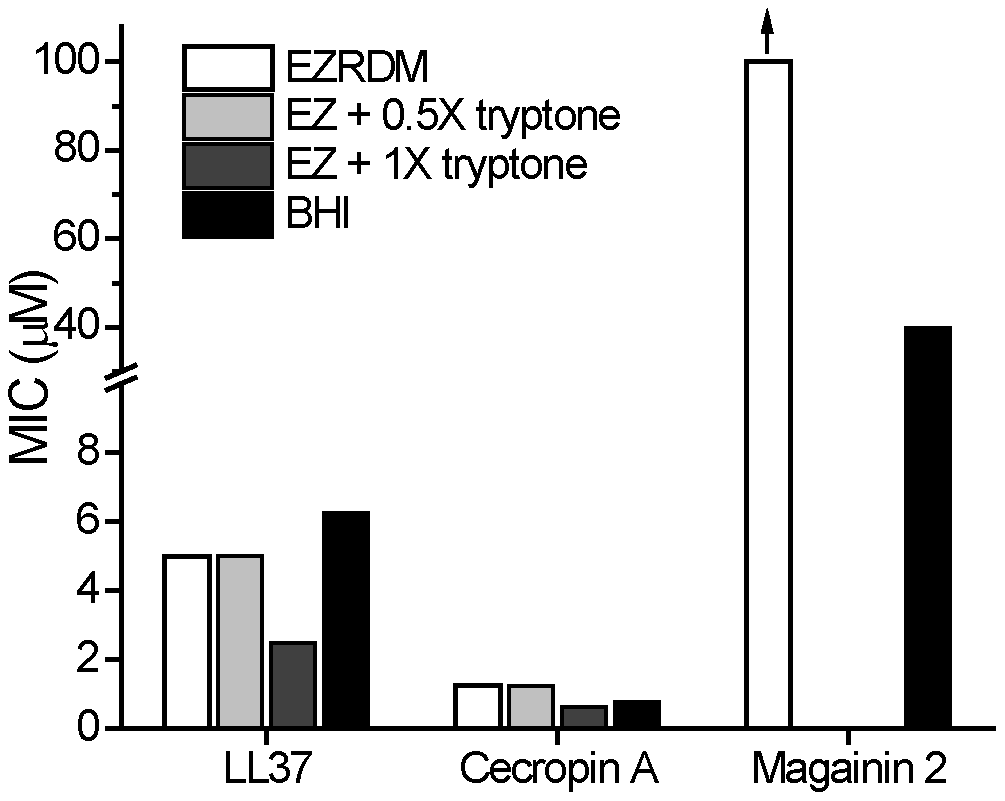
Minimum inhibitory concentrations (MICs) of three natural antimicrobial peptides, LL-37, Cecropin A, and Magainin 2, in different media. EZRDM and BHI as described in text. See Fig. 5 for sequences of the antimicrobial peptides. Note break in vertical scale for Magainin 2. The designations “EZ + 0.5X tryptone” and “EZ + 1X tryptone” refer to EZRDM supplemented with 5 g/L, and 10 g/L of dialyzed tryptone powder, respectively. Vertical arrow marks a bar that is a lower limit only.

For comparison, we measured the MIC values against *E. coli* of two natural antimicrobial peptides, LL-37 and Cecropin A, in four media: BHI, EZRDM, and EZRDM supplemented with 0.5X or 1X tryptone. As shown in Fig. 3, LL-37 and Cecropin A showed strong antimicrobial activity in all four media. Detailed OD data are provided in Fig. S4. In fact, MIC values for these two AMPs were slightly smaller in EZRDM plus 1X tryptone than in unsupplemented EZRDM. The natural AMP Magainin 2 was not active against *E. coli* in EZRDM (MIC>100 µM) and had an MIC of 40 µM in BHI. Unlike polymers **A–C**, Magainin 2 does not gain activity when the medium changes from BHI to EZRDM (Fig. 3).

## Discussion

### Medium Effects on polymer MIC values

The susceptibility of bacteria to antibiotics, including antimicrobial peptides, can depend on environmental conditions such as temperature, aeration, and pH, as well as the concentrations of ionic species [4]. A high concentration of Ca^2+^ [27] or Mg^2+^ [28] in the growth medium increases MIC values for AMPs, presumably due to competition between the divalent cation and the cationic peptide for binding sites within the bacterial cell surface. Addition of polyanions such as DNA [29] also increases MIC values, presumably by binding to cationic AMPs and decreasing the concentration of free AMP available for disruptive interactions with the bacterial cell surface. To avoid such complications, most laboratories screen for antimicrobial peptide activity without varying the growth medium. The few studies that compare MIC values between two different media [30], [31] have not provided clear conclusions because of the inherent complexity of the broths employed. The present work compares cationic polymer and AMP activities in two widely used complex media (BHI and LB) with activities in a chemically defined medium (EZRDM). This experimental design enables us to draw some tentative conclusions about the underlying causes of the strong variation in MIC values across media.

Our primary finding is that BHI (which usually contains peptone supplement) and LB (which contains tryptone supplement) suppress polymer activity against *E. coli* as compared with EZRDM (which contains only small molecules). By adding 1X dialyzed tryptone solution to EZRDM, we were able to recapitulate polymer performance in BHI and LB. To avoid possibly confounding effects of small molecules such as salts, sugar, vitamins, and divalent cations, we used material derived from tryptone powder via dialysis vs water for several days, followed by lyophilization. We therefore attribute the effects of adding tryptone to EZRDM to the polypeptides that are generated via tryptic digestion of casein.

**Figure 4:**
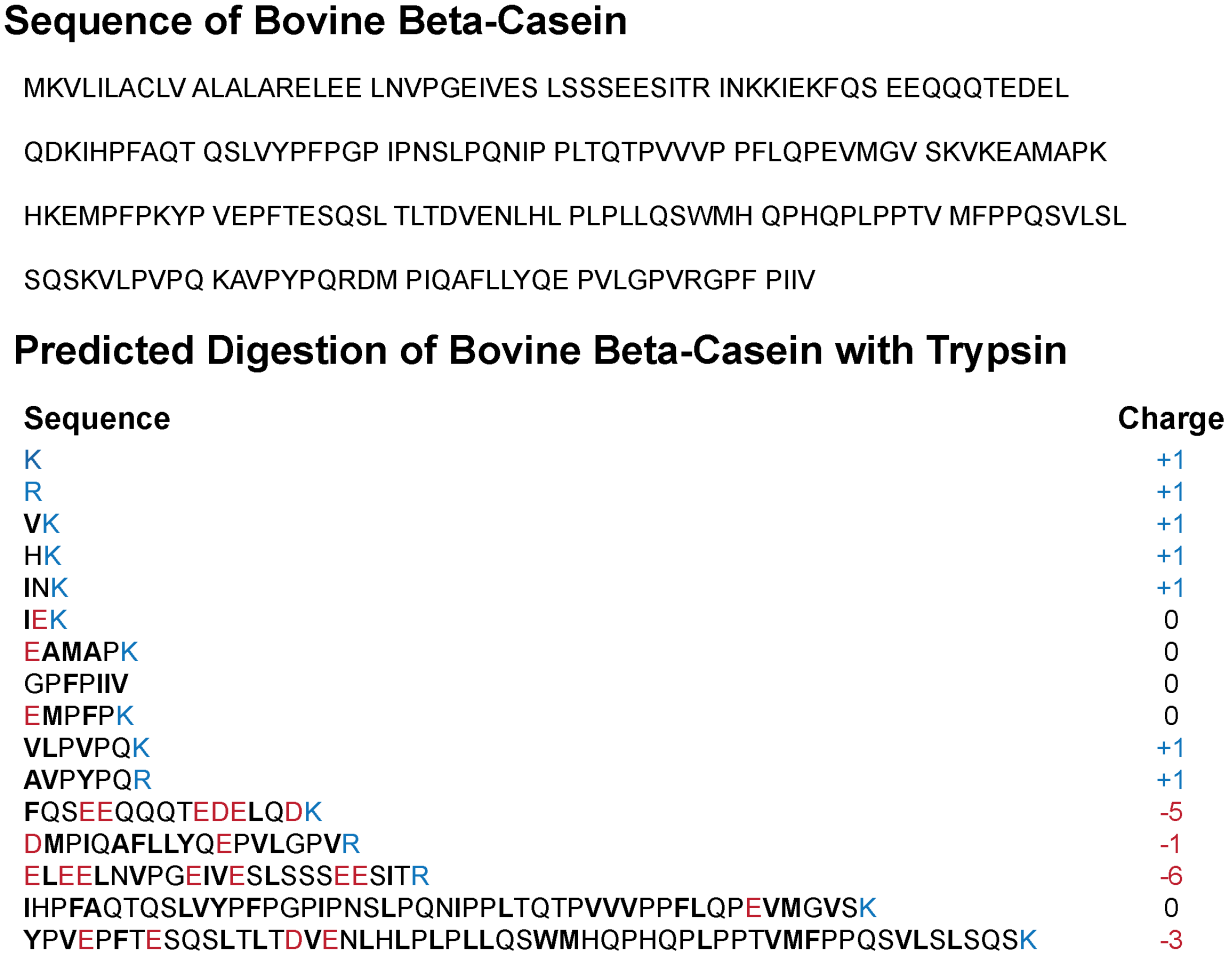
Sequence of bovine beta-casein and of the predicted components of complete digestion of bovine β-casein with trypsin. Red, blue, and bold-black letters denote anionic, cationic, and hydrophobic residues, respectively. Net charges as shown.

What are these polypeptides? Trypsin cleaves peptide bonds specifically at the C-terminal side of lysine and arginine residues. Complete digestion necessarily produces peptides with only one positive charge (at the C-terminus). Digest products are thus intrinsically biased to be negatively charged or hydrophobic or both. The sequence of bovine casein and the predicted products of its complete digestion by trypsin are shown in Fig. 4. This predicted mixture includes six peptides containing 1–7 residues, which have a net charge of +1 or are neutral; such small peptides are presumably depleted from raw tryptone solution during dialysis. In addition, there are five longer peptides, including three that contain 16–24 residues (net charge ranging from −1 to −6), one with 48 residues (neutral) and one with 56 residues (net charge −3). Highly anionic components include 16-mer FQSEEQQTEDELQDK (net charge −5), and 24-mer ELE…, with net charge −6. The two longest peptides are highly hydrophobic.

Supplementation of EZRDM with 1X tryptone causes a profound diminution in the ability of polymers **A**–**C** to inhibit growth of *E. coli* (Fig. 2). We propose that all three polymers bind to the polyanionic peptides generated via tryptic digestion of casein. The resulting polymer-anionic peptide complexes presumably bind less strongly to the anionic lipopolysaccharide (LPS) layer found on the outer surface of *E. coli* than do uncomplexed polymers. Even for polymer **A**, which displays significant activity in BHI, the MIC decreases fourfold from EZRDM plus 1X tryptone to EZRDM or from LB to EZRDM, and eightfold from EZRDM to BHI from EZRDM.

This hypothesis is further supported by the MIC pattern of the three polymers in EZRDM supplemented with anion-exchanged tryptone, which is quite similar to the MIC pattern in EZRDM alone (Fig. 2). Addition of the anionic peptide FQSEEQQTEDELQDK, which is predicted to be generated by digestion of beta-casein with trypsin, increased the MICs of the three polymers by a factor of four to eight (Fig. 2). Addition of this peptide did not, however, match the full tryptone-induced increases in MICs for polymers **B** and **C**. This finding with a specific peptide supports our hypothesis regarding the MIC-suppressing effects of polyanionic components on a polymer's antibacterial activity. However, this observation also suggests that the interactions between constituents of the polyanion mixture in tryptone and a heterogeneous cationic polymer sample may be difficult to understand in detail.

There is precedent for our observation that the antibacterial effect of highly cationic polymers can be sensitive to the growth medium. The cationic homopolymer ε-polylysine is used as a food preservative based on its antibacterial effects [32]. The reported MIC for ε-polylysine against *E. coli* decreases from 50 µg/mL [33] to 1 µg/mL [34] (50-fold) when the growth medium is changed from nutrient buffer (which includes peptone) to Davis medium (which is a chemically defined medium containing “casamino acids”, a mixture of *monomeric* α-amino acids). Since the ingredients of Davis medium do not include a *peptide*-rich component generated via enzymatic degradation of proteins, such as peptone or tryptone, the pronounced effect of medium on the antibacterial activity of ε -polylysine is consistent with our conclusions about the antibacterial activity of cationic nylon-3 polymers. Our results may also be relevant to studies using cationic peptides to enhance the permeability of the outer membrane to hydrophobic drugs [35].

### Implications for the design of antimicrobial polymers

The three polymers evaluated here were selected based on a previous study of structure-function relationships within the nylon-3 family [19]. That study measured MIC (in BHI medium) and minimum hemolytic concentration (MHC) values for polymers that varied in the nature of the hydrophobic and cationic subunits, the ratio of hydrophobic to cationic subunits, the N-terminal group, and the mean chain length. The composition embodied in polymer **A**, at ∼25-mer average chain length, exhibited the best performance overall. This nylon-3 polymer displayed moderate MIC values against both Gram negative and Gram positive bacteria along with a high MHC value. Analogous polymer **B**, which lacks the hydrophobic unit at the N-terminus, was far less active than **A** against *E. coli*. Homopolymer **C** also was far less active than **A** against *E. coli*, which indicated that hydrophobic CH subunits are critical for conferring antibacterial activity on polymer **A**. The trends previously observed among nylon-3 polymers **A**–**C** are consistent with conclusions drawn from studies of natural AMPs and analogous synthetic peptides, which suggest that optimizing antibacterial activity while simultaneously minimizing eukaryotic cell toxicity (e.g., hemolytic activity) requires the presence of both hydrophobic and cationic subunits, with a proper balance between net charge and net lipophilicity [36].

The previous nylon-3 studies were conducted in BHI medium. The present results show that the impact of introducing hydrophobic subunits or cationic N-terminal groups can be dramatically altered by changing the medium. We observe that all three nylon-3 polymers are more active against *E. coli* (lower MIC values) in EZRDM than in BHI medium. Most striking is the observation that cationic homopolymer **C** is highly active in EZRDM, while this polymer has very little activity against *E. coli* in BHI medium.

Our study raises the general question of which media are most appropriate for evaluating the activity of new antimicrobial candidates. The ε-polylysine precedent [32] and the sensitivity to medium we document for highly cationic polymers suggest that investigations in chemically defined media have substantial merit for such evaluations. In addition, many pathogenic bacteria are more virulent in minimal growth conditions than in rich growth conditions [37], [38], a trend that argues for using a chemically defined *minimal* medium to evaluate the antimicrobial activities of new polymers.

**Figure 5:**
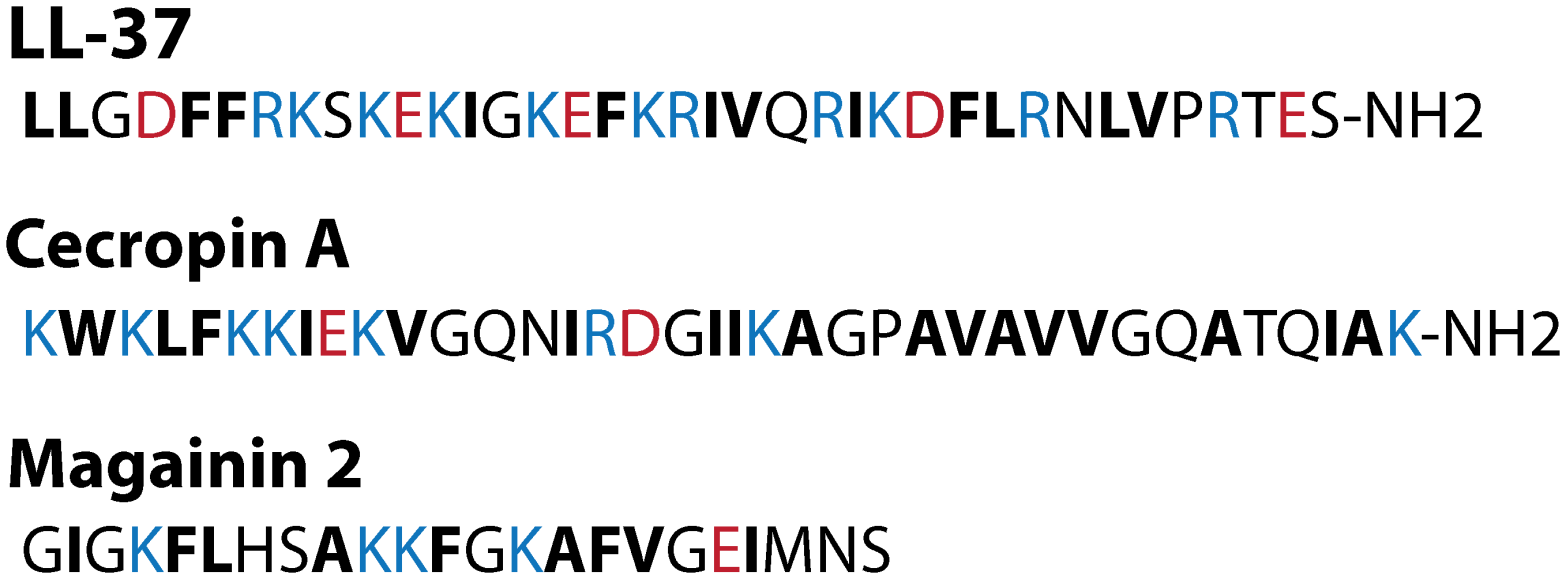
Sequences of the natural AMPs LL-37, Cecropin A, and Magainin 2. Red, blue, and bold-black letters denote anionic, cationic, and hydrophobic residues, respectively.

The natural cationic AMPs LL-37 and Cecropin A are remarkably impervious to the medium effects that we find to be so strong for the highly cationic polymers (Fig. 3). AMPs may have evolved so as to maintain their efficacy in a variety of environments. The sequences of LL-37 and Cecropin A (Fig. 5) are perhaps instructive. The positive charge density is smaller, on a per-subunit basis, in the natural AMPs than in nylon-3 polymers **A-C** because the peptides contain residues that are neither positively charged nor hydrophobic. The lower positive charge density and the inclusion of some negatively charged residues may diminish binding of the natural AMPs to polyanionic species in BHI and in tryptone-supplemented EZRDM. This speculation suggests that it may prove fruitful to study random polymers comprising three or even four components, with proportions of cationic, anionic, and hydrophobic monomers chosen to mimic those found in natural AMPs. Unlike the nylon-3 polymers, which gained activity when moved from BHI to EZRDM, Magainin 2 showed low activity (MIC = 40 µM) in BHI and no activity (MIC>100 µM) in EZRDM (Fig. 3). Magainin 2, with 23 residues and a net charge of +3 at neutral pH, may not be sufficiently cationic to bind effectively to the LPS layer of Gram negative species.

It is possible that AMPs and highly positive nylon-3 polymers attack bacterial cells by different mechanisms, and that the mechanism of action varies among the nylon-3 polymers. Many AMPs fold into amphipathic helices and may form membrane pores of reasonably well defined structure. Defined conformations are not available to the sequence-random copolymers or the purely cationic polymers studied here. It is not clear how a highly cationic polymer such as **C** could cause membrane disruption, which evidently underlies the antibacterial effects of peptides such as LL-37 and Cecropin A [23]–[25]. The observation that diverse nylon-3 polymers display strong growth-inhibitory activity toward *E. coli* in EZRDM will enable detailed investigations of the mechanism(s) of action via optical imaging methods.

## Supporting Information

Figure S1 Detailed data behind MIC measurements for the polymers and the antimicrobial peptides for different media and conditions are provided as supporting information.
**Optical density (O.D.) vs concentrations of two different batches of polymers in BHI medium.**
(TIF)

Figure S2 **Optical density (O.D.) vs concentration compared for two different media and two different** ***E. coli*** **strains.**
(TIF)

Figure S3 **MIC measurements in different media as indicated.** (A) Optical density (O.D.) vs concentration of polymers **A**, **B**, and **C** in different media. (B) Bar graph of MIC values.
(TIF)

Figure S4 **Optical density (O.D.) vs concentration of natural antimicrobial peptides Magainin 2, Cecropin A, and LL-37 for different media.**
(TIF)

Figure S5 **Minimum inhibitory concentrations (MICs) of nylon-3 polymers A, B, and C in EZRDM supplemented with 200 µM and 400 µM of the anionic peptide FQSEEQQTEDELQDK.**
(TIF)
