# Correction: Medium Effects on Minimum Inhibitory Concentrations of Nylon-3 Polymers against *E. coli*

**DOI:** 10.1371/journal.pone.0116241

**Published:** 2014-12-17

**Authors:** The PLOS ONE Staff

There are errors in Figure 2 of the published paper. The correct Figure 2 can be viewed here.

**Figure 2:**
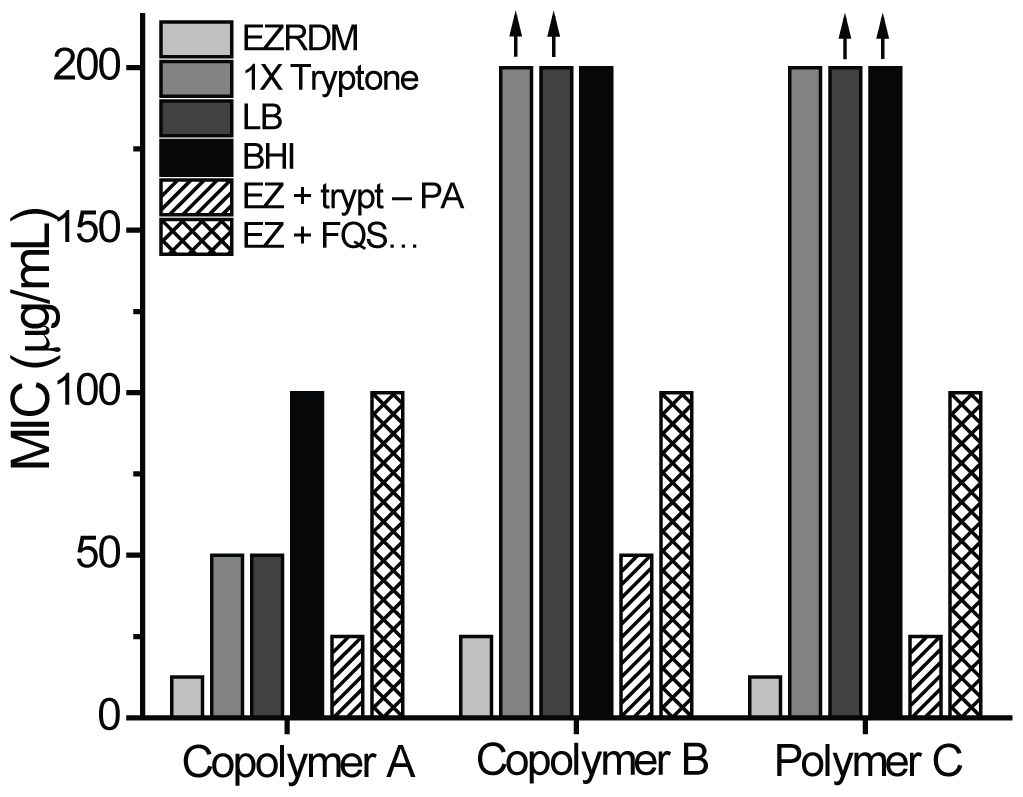
Minimum inhibitory concentrations (MICs) of three nylon-3 polymers against *E. coli* for different media. See Fig. 1 for structures of A, B, and C. Vertical arrows mark bars that are lower limits only. EZRDM, LB, and BHI as described in main text. The designation “EZ + trypt” refers to EZRDM supplemented with 10 g/L of dialyzed tryptone powder (1X tryptone). “EZ + trypt – PA” refers to EZRDM supplemented with anion-exchanged tryptone at the equivalent of 10 g/L. “EZ + FQS…” refers to EZRDM supplemented with 400 µM of the single anionic peptide FQSEEQQTEDELQDK (net –5 charge). doi:10.1371/journal.pone.0104500.g002
