# Supplementary figures and images for: Medium Effects on Minimum Inhibitory Concentrations of Nylon-3 Polymers against E. coli
Source: PLoS One. 2014 Aug 25;9(8):e104500. doi: 10.1371/journal.pone.0104500 (PMC4143223; doi:10.1371/journal.pone.0104500)

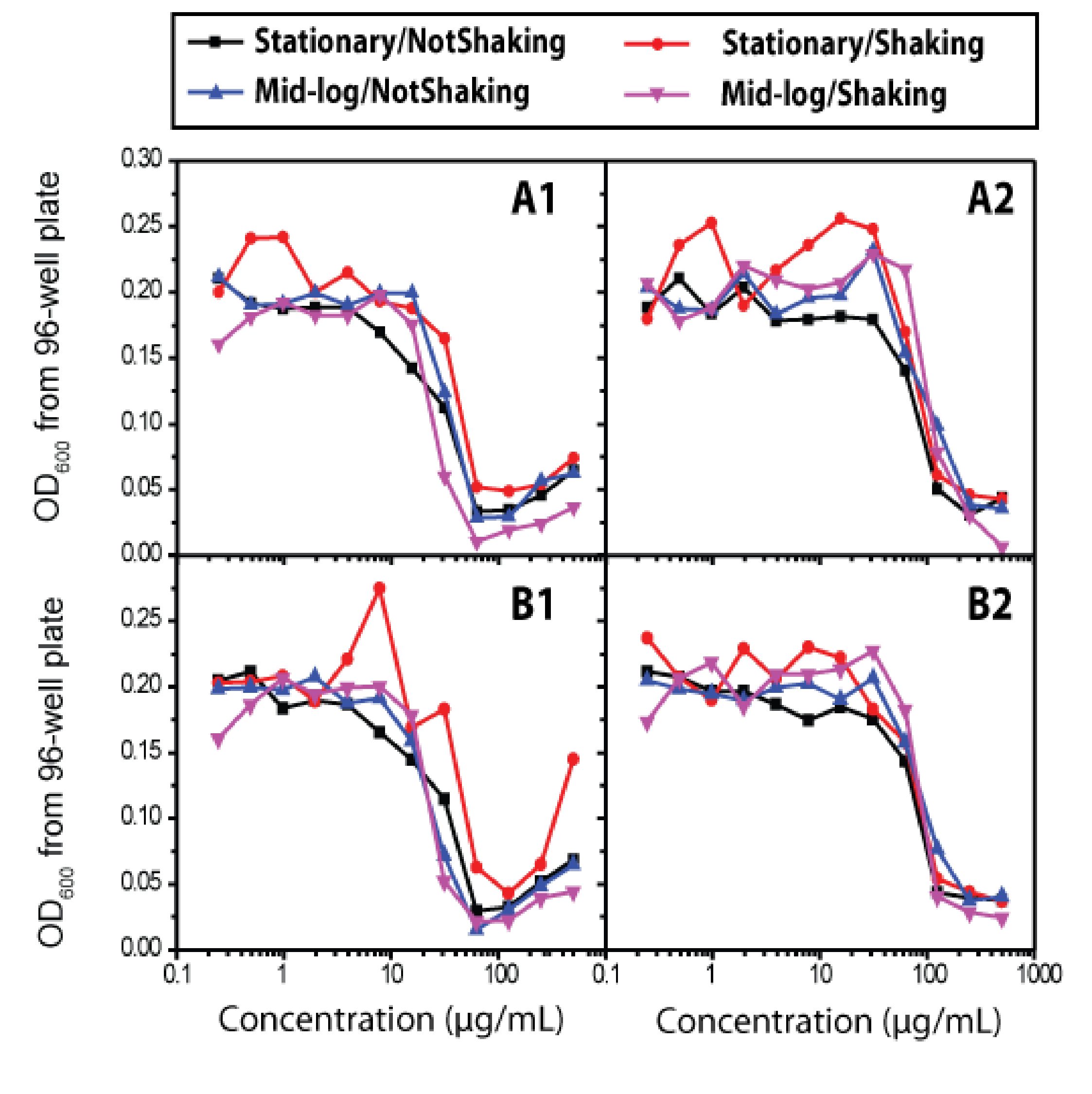

Supplement: Figure S1 — Detailed data behind MIC measurements for the polymers and the antimicrobial peptides for different media and conditions are provided as supporting information. Optical density (O.D.) vs concentrations of two different batches of polymers in BHI medium. (TIF) [file pone.0104500.s001.tif]

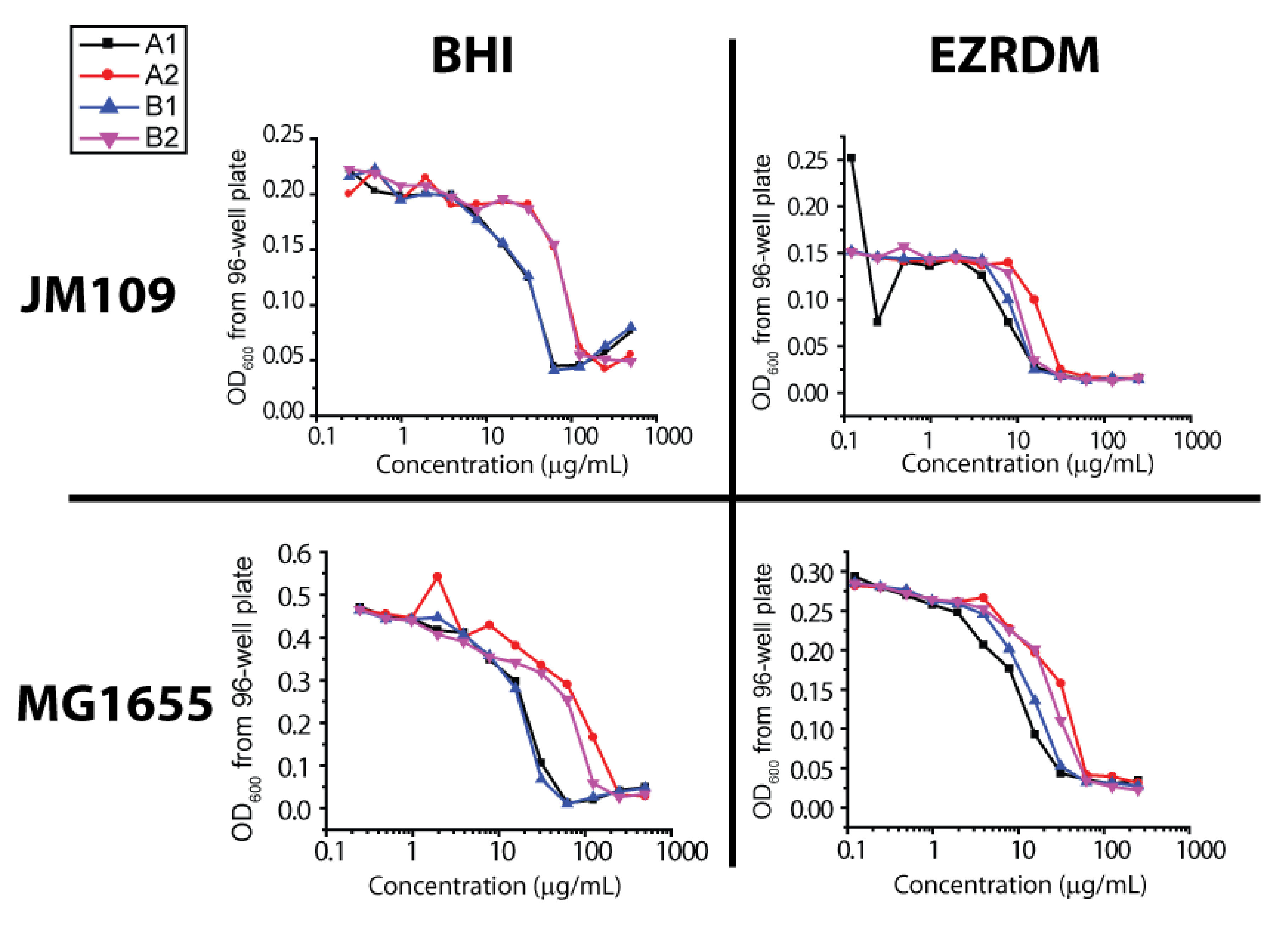

Supplement: Figure S2 — Optical density (O.D.) vs concentration compared for two different media and two different E. coli strains. (TIF) [file pone.0104500.s002.tif]

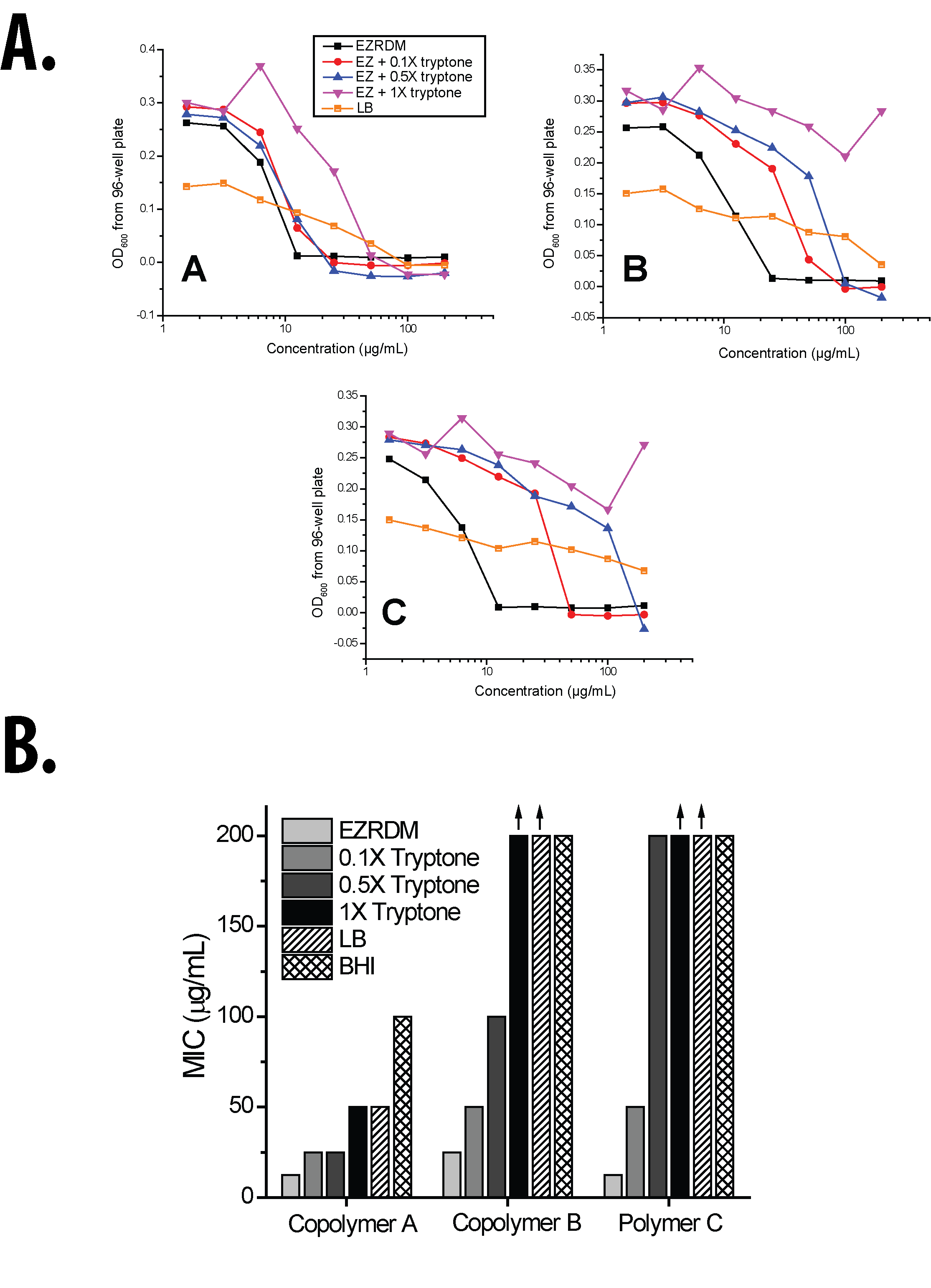

Supplement: Figure S3 — MIC measurements in different media as indicated. (A) Optical density (O.D.) vs concentration of polymers A, B, and C in different media. (B) Bar graph of MIC values. (TIF) [file pone.0104500.s003.tif]

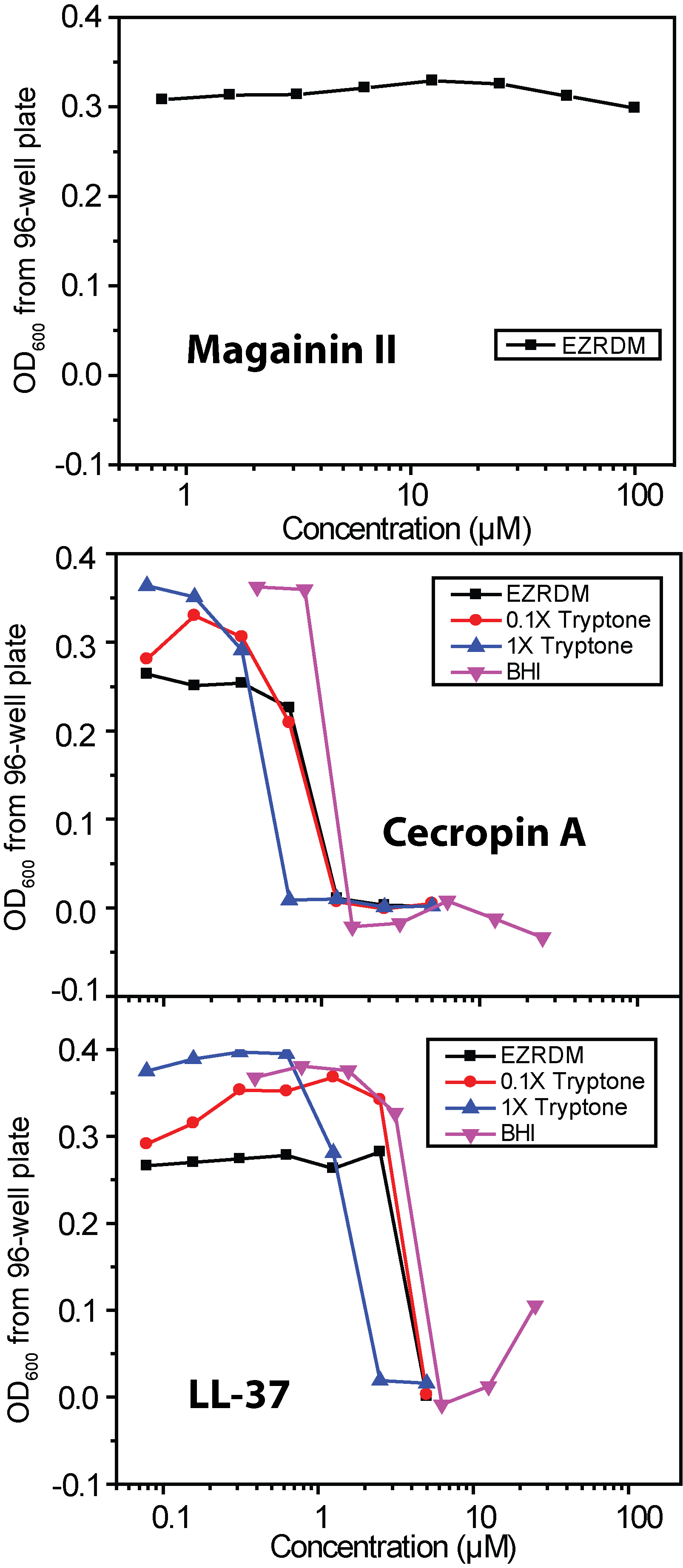

Supplement: Figure S4 — Optical density (O.D.) vs concentration of natural antimicrobial peptides Magainin 2, Cecropin A, and LL-37 for different media. (TIF) [file pone.0104500.s004.tif]

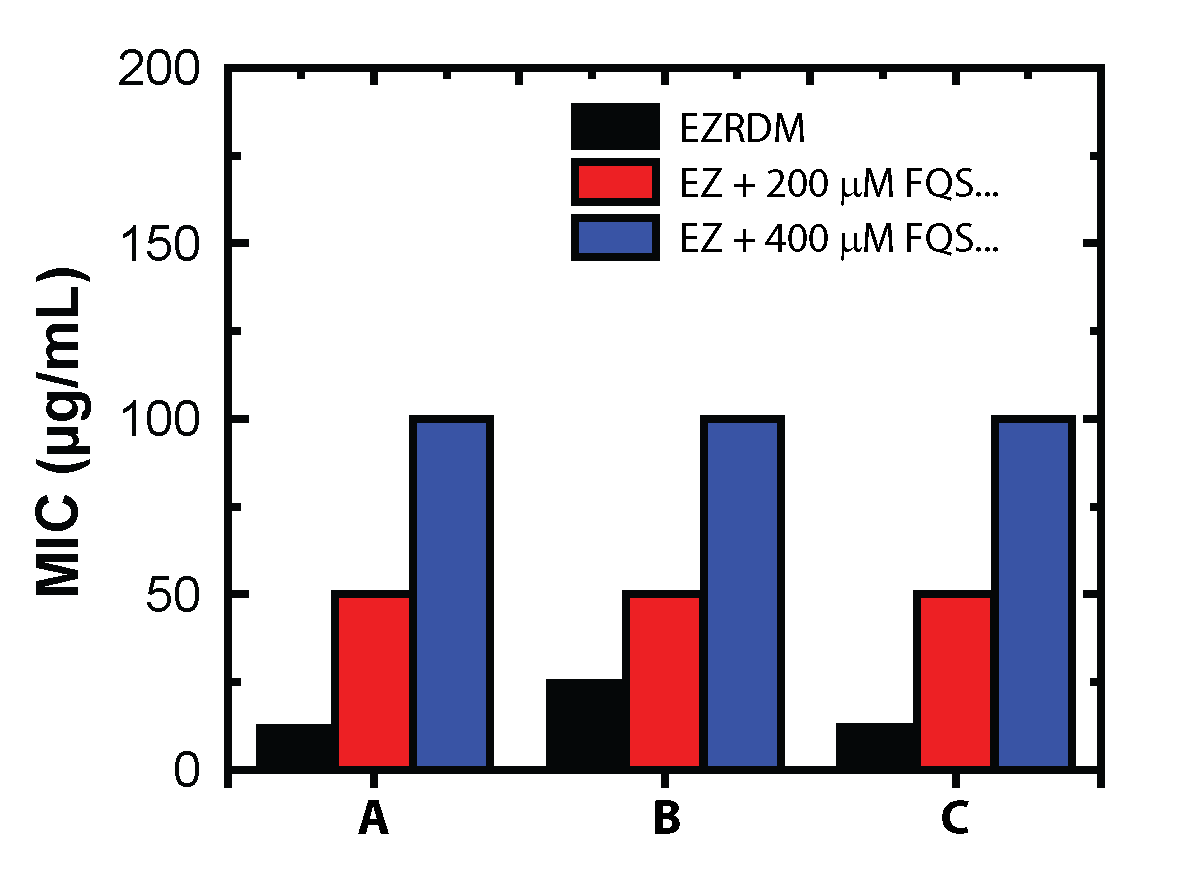

Supplement: Figure S5 — Minimum inhibitory concentrations (MICs) of nylon-3 polymers A, B, and C in EZRDM supplemented with 200 µM and 400 µM of the anionic peptide FQSEEQQTEDELQDK. (TIF) [file pone.0104500.s005.tif]
